# The Wilms’ tumor gene (WT1) regulates E-cadherin expression and migration of prostate cancer cells

**DOI:** 10.1186/1476-4598-12-3

**Published:** 2013-01-08

**Authors:** Adina Brett, Sony Pandey, Gail Fraizer

**Affiliations:** 1 School of Biomedical Sciences, Kent State University, Kent, OH, 44242, USA; 2 Department of Biological Sciences, Kent State University, Kent, OH, 44242, USA

**Keywords:** WT1, E-cadherin, Prostate cancer, Migration, Metastasis

## Abstract

**Background:**

One key step in the development of prostate cancer (PCa) metastasis is the loss of E-cadherin expression associated with increased cellular motility and tumor invasion. This loss of E-cadherin expression is also required during normal embryogenesis and similar transcriptional repressors have been identified in both processes. We have previously reported the presence of one such transcription factor, WT1 in high Gleason grade prostate tumor tissues, and its absence in non-neoplastic or benign prostatic hyperplasia tissues.

**Results:**

To better understand the effect of WT1 on E-cadherin expression and migration of PCa cells we quantified WT1 and E-cadherin mRNA levels in normal prostate epithelial and PCa cell lines with varying migratory potential. In WT1 transfected cells E-cadherin transcript levels were decreased, while they were increased in siWT1-RNA transfected PCa cells, suggesting that elevated WT1 expression was sufficient to dampen E-cadherin levels and potentially enhance migratory ability. To delineate the mechanism of WT1-mediated repression of E-cadherin, potential WT1 binding sites were tested *in vitro* and *in vivo* binding of WT1 to the E-cadherin promoter in the chromatin of LNCaP and PC3 cells was assessed by Chromatin Immunoprecipitation. The effect of WT1 binding was measured in reporter assays; in PC3 and DU145 cells WT1 decreased the activity of the proximal E-cadherin promoter. Using site-directed mutagenesis, a newly identified WT1 binding site located 146 bp from the transcription start site was shown to be required for this repression by WT1. Transwell migration and wound healing assays revealed that in LNCaP cells with low migratory potential, over-expression of WT1 was sufficient to enhance migration, conversely, in the highly migratory PC3 cells silencing of WT1 decreased migration.

**Conclusions:**

These findings suggested that WT1 expression in high grade prostate cancer may contribute to migration and metastasis. Thus, in prostate cancer WT1 may function as a novel oncogene facilitating development of the lethal metastatic phenotype.

## Background

Prostate cancer (PCa) is the second leading cause of cancer death among men in the USA [1]. Although patients with localized prostate cancer have high survival and relative low mortality rate, patients with detectable metastases have median survival of 12–15 months, suggesting that metastatic process is the main cause of high mortality among PCa patients [2]. The loss of cell adhesion is a crucial step in the process of metastasis and a critical early event is the conversion of the stationary to the migratory phenotype [3]. When cancer cells acquire motility and invasiveness, they undergo drastic changes in their structure: lose epithelial features, such as adhesion, and acquire a more mesenchymal phenotype. This modification of cancer cells is known as the epithelial to mesenchymal transition (EMT) [4,5] and the loss of cell-cell interaction is caused by suppression of cell adhesion molecules such as E-cadherin, normally expressed by epithelial cells. Loss or down regulation of E-cadherin expression is associated with an increase in the migration and invasiveness of many types cancer cells [6] including prostate [7-10].

Down regulation of E-cadherin has been reported to occur via hypermethylation [11,12], mutations [13] and transcription factors such as Snail [14,15], Slug [16], ZEB-1 and ZEB-2(SIP-1) [17] and the bHLH family of factors E12/E47 and Twist [18,19]. E-cadherin has also been shown to be regulated by the zinc finger transcription factor WT1 in NIH3T3 fibroblasts [20] and in cardiac epithelial cells undergoing EMT, WT1 represses E-cadherin expression both directly and indirectly by the upregulation of the repressor Snail [21]. Although multiple isoforms of WT1 have been identified, those that regulate transcription lack a tripeptide (KTS) insertion in exon 9 [22]. Our study focuses on identifying the function of the transcriptionally active isoform lacking both exon 5 and KTS in prostate cancer cells. WT1 was first identified as a tumor suppressor based on its mutational inactivation in Wilms’ tumors of the kidney [23]. In contrast, in other tumor types, WT1 levels are elevated, suggesting an oncogenic role [24-30]. In PCa, it has been reported that WT1 is a marker of human PCa progression [29], and that WT1 is primarily expressed in high Gleason grade PCa epithelial cells [30]. However the relationship of WT1 to E-cadherin expression in PCa has not been characterized.

Here we tested the effect of WT1 on endogenous E-cadherin mRNA levels by both silencing and overexpressing WT1. Potential WT1 binding sites in the E-cadherin promoter were characterized using chromatin immunoprecipitation (ChIP) and site-directed mutagenesis. The biological impact of WT1-mediated repression of E-cadherin was tested by migration and wound healing assays in PCa cells with varying migratory potential. Overall, this study was designed to determine whether WT1 transcriptionally regulated E-cadherin and thereby, migration of PCa cells.

## Results

### Transcriptional repression of E-cadherin is mediated through a novel WT1 binding site in the E-cadherin proximal promoter

**Figure 1:**
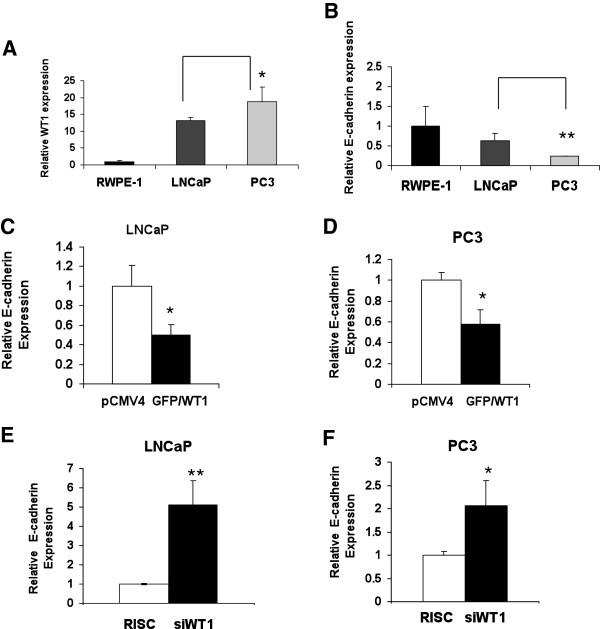
**WT1 and E-cadherin mRNA expression in prostate cancer (PCa) cells.** RNA was isolated from RWPE-1, LNCaP and PC3 cells, reverse transcribed and (**A**) WT1 and (**B**) E-cadherin transcript levels were measured using TaqMan qRT-PCR. Values were normalized to 18S transcripts levels and data are presented relative to the non-neoplastic prostate epithelial cell line RWPE-1. Experiments were done in triplicate and reproduced twice. Student t-test was performed and significance was determined by comparing expression of WT1 (**A**) and E-cadherin (**B**) in PC3 cells with that of LNCaP cells (*p ≤ 0.05, **p ≤ 0.01). (**C** and **D**) E-cadherin mRNA expression levels in LNCaP (**C**) and PC3 (**D**) PCa cells transfected with pCMV4 or GFP/WT1 expression construct were measured using TaqMan qRT-PCR. Values were normalized as described in (**A** and **B**). Data are presented relative to pCMV4 transfected cells. Experiments were done in triplicate and reproduced three times. Student t-test was performed and significance was determined by comparing pCMV4 to GFP/WT1 transfected cells (*p ≤ 0.05). (**E** and **F**) E-cadherin mRNA expression in LNCaP (**E**) and PC3 (**F**) PCa cells transfected with RISC or siWT1 RNA oligonucleotides were measured as described in (**A** and **B**). Data are presented relative to RISC transfected cells. Significance was determined by Student t-test comparing RISC to siWT1 RNA oligonucleotides transfected cells (*p ≤ 0.05, **p ≤ 0.01) in three independent experiments.

Previous studies in our lab demonstrated differences in WT1 mRNA levels between normal prostate epithelial (RWPE-1) and PCa cell lines [30] and others have reported that E-cadherin protein levels were higher in LNCaP than PC3 [31]. Thus, we analyzed whether WT1 and E-cadherin mRNA levels were inversely related in these cell lines. Quantitative real-time PCR (qRT-PCR) was performed to measure the levels of WT1 and E-cadherin mRNA in these PCa cell lines. As expected, we found that WT1 (Figure 1A) and E-cadherin (Figure 1B) mRNA levels were inversely related. This inverse relationship is consistent with reports of others that WT1 is a regulator of E-cadherin in NIH 3T3 [20] and epicardial cells [21]. To determine whether WT1 is a regulator of E-cadherin gene expression in LNCaP and PC3 PCa cells, we transfected the cells with pCMV4 or GFP/WT1 expression vector and measured the effect of WT1 overexpression by TaqMan qRT-PCR as described below. Our results showed that GFP/WT1 transfection reduced E-cadherin mRNA levels 2-fold in LNCaP (Figure 1C) and 1.7-fold in PC3 (Figure 1D) cells compared with pCMV4 transfected cells. These results were reproduced thrice and consistently showed that increased WT1 expression (≥10-fold) is sufficient to repress E-cadherin mRNA levels. To determine the effect of decreased WT1 levels on E-cadherin regulation, we transfected LNCaP and PC3 cells with scrambled (RISC) or targeted siWT1 RNA oligonucleotides (Dharmacon) or no RNA (MOCK). The levels of WT1 and E-cadherin mRNA were measured using TaqMan qRT-PCR. Transfection with siWT1 RNA oligonucleotide #7 (Dharmacon) reduced WT1 mRNA levels by 88% in PC3 and 81% in LNCaP cells (data not shown). The suppression of WT1 expression increased the levels of E-cadherin mRNA 5-fold in siWT1 RNA oligonucleotide transfected LNCaP (Figure 1E), and 2-fold in PC3 cells (Figure 1F) cells compared to the scramble oligonucleotides (RISC). These results were reproduced in three independent experiments with similar findings. Additionally, results with oligonucleotide #7 (Dharmacon) were confirmed using a differently targeted siWT1 RNA oligonucleotide, #8 (Dharmacon) that also similarly decreased WT1 mRNA levels by 81% and increased E-cadherin mRNA levels in PC3 cells by 1.9-fold (data not shown). Surprisingly WT1 protein levels were less strongly affected by siRNA oligonucleotides and thus E-cadherin protein levels were only modestly increased (data not shown). Overall, these results showed that E-cadherin transcript levels were inversely affected by WT1 over-expression and siWT1 RNA knock-down in these two PCa cell lines.

**Figure 2:**
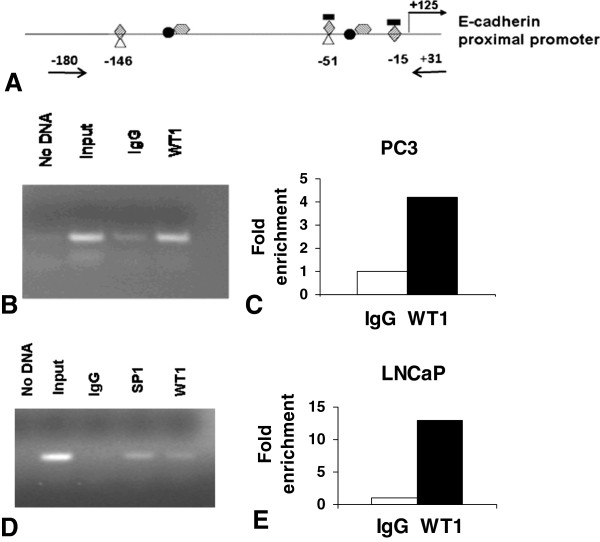
**WT1 binds to E-cadherin promoter*in vivo.*** (**A**) Schematic diagram of E-cadherin promoter with transcription factors potential binding sites: WT1  EGR-1 , Snail , Twist , SP1 . Positions of potential WT1 binding sites are listed and arrows indicate the location of PCR primers used for amplification of chromatin. ChIP assays were performed with chromatin from PC3 (**B**) and LNCaP (**D**) cells. Cells were transfected with GFP/WT1 construct and harvested after 48 hours. Chromatin was crosslinked and then immunoprecipitated with either IgG (negative control), WT1 (**B**, **D**) or SP1 (positive control) (**D**) antibody. Input or immunoprecipitated DNA was amplified by endpoint PCR, as described in Methods, using primers that amplify a 210 bp region of the E-cadherin proximal promoter. (**B** and **D**) Amplified products were analyzed by gel electrophoresis and representative images are shown. (**C** and **E**) Sybergreen qRT-PCR was performed to quantify the WT1 immunoprecipitated DNA from PC3 (**C**) or LNCaP (**E**) cells. Experiments were reproduced twice with different chromatin preparations and representative qRTPCR results are shown as fold enrichment compared to IgG.

To determine how WT1 might regulate E-cadherin expression we identified potential WT1 binding sites in the E-cadherin promoter, using the MatInspector software. We identified two potential WT1 binding sites at −15 and −146 bp upstream from the transcription start site (Figure 2A). A third WT1 binding site at −51 bp was previously reported functional in fibroblasts [20] but not identified by the MatInspector software (Figure 2A). Moreover, two potential EGR-1 binding sites at −15 and −55 and two potential Twist binding sites at −37 and −87 bp upstream from the start site were predicted by the same software. Additionally, other binding sites in the E-cadherin promoter for transcription factors such as Snail have been reported [32]. The two predicted EGR-1 binding sites overlap with WT1 sites at −15 and -51bp as do two SP1 binding sites previously reported at −51 and −146 bp [32]. Multiple overlapping WT1, EGR-1 and SP1 binding sites were predicted in promoter regions of genes expressed in PCa epithelial cells [33]. Our previous bioinformatics studies also identified an evolutionarily conserved region containing a potential overlapping WT1 and EGR-1 binding site in the E-cadherin promoter [33]. To test whether the region of the E-cadherin promoter containing WT1 sites was functional in PCa cells, Chromatin Immunoprecipitation (ChIP) was performed. LNCaP and PC3 cells were transfected with the pGFP/WT1 expression construct [34] to increase the levels of –KTS isoform and enhance potential binding to the native chromatin of LNCaP and PC3 cells. Chromatin of PC3 (Figure 2B) and LNCaP (Figure 2D) cells was immunoprecipitated with anti-WT1 antibodies and amplified by endpoint PCR using primers (Figure 2A) designed to flank the regulatory region of the E-cadherin gene containing three WT1 potential binding sites. Additionally Sybergreen (Agilent, La Jolla, CA) quantitative real-time PCR (qRT-PCR) analysis of the E-cadherin promoter region was performed to validate and quantify representative samples of immunoprecipitated chromatin from PC3 (Figure 2C) and LNCaP (Figure 2E) cells. Additional chromatin preparations from both cell lines were also analyzed for confirmation of binding initially using the endpoint PCR method and then validating by qRT-PCR with similar results (data not shown). Specificity of WT1 binding was assessed by endpoint PCR using primers that flanked a region lacking potential WT1 binding sites, located ~1kb upstream of the transcriptional start site (Additional file 1: Figure S1). These results demonstrate specific *in vivo* DNA binding by WT1, a prerequisite for WT1 mediated regulation of the E-cadherin gene expression in PCa cells.

**Figure 3:**
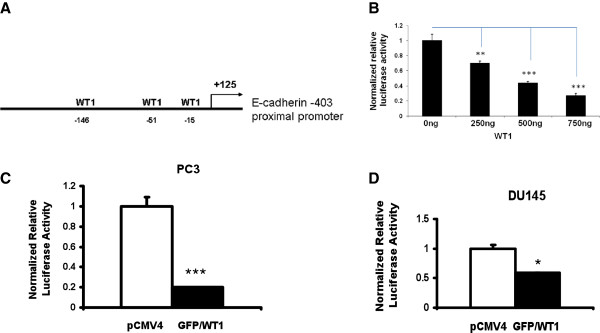
**WT1 represses E-cadherin proximal promoter.** (**A**) Schematic diagram of the E-cadherin proximal promoter with 3 potential WT1 binding sites. (**B**) Effect of WT1 on E-cadherin promoter was tested in a dose response in PC3 cells. 250 ng of the E-cadherin proximal promoter reporter construct was cotransfected either with pCMV4 or GFP/WT1 expression construct at 0, 250, 500 and 750 ng concentrations. DNA concentrations were held constant by adding increasing amounts of pCMV4 and reporter activity analyzed as described in methods. (**C** and **D**) E-cadherin proximal promoter reporter construct was cotransfected either with pCMV4 or GFP/WT1 expression construct in PC3 (**C**) and DU145 (**D**) cells. Luciferase activity was measured and normalized to protein concentration. Data are reported relative to luciferase activity of pCMV4 transfected cells. Experiments were repeated three times in triplicate. Significance was determined by student’s t-test comparing GFP/WT1 transfected cells to pCMV4 transfected (*p ≤ 0.05, ***p ≤ 0.001).

To determine whether WT1 transcriptionally regulates E-cadherin promoter activity, a reporter construct containing the region 403 bp upstream of the E-cadherin transcription start site was cloned from genomic DNA, as described in methods. To analyze the effect of overexpression of WT1 on the E-cadherin proximal promoter, the E-cadherin reporter construct (Figure 3A) was co-transfected along with increasing doses of GFP/WT1 expression construct in PC3 cells and luciferase activity was measured as described in methods. As shown in Figure 3B, WT1 repressed the E-cadherin proximal promoter in a dose dependent manner, with 500 ng of GFP/WT1 achieving a greater than 50% reduction of the promoter activity. These results together with gene expression studies, suggested that WT1 mediated repression of E-cadherin could maintain low levels of expression of E-cadherin in PCa cells. To confirm the effect of WT1 overexpression on the E-cadherin proximal promoter, the reporter construct was transiently co-transfected along with GFP/WT1 expression construct in both PC3 (Figure 3C) and DU145 (Figure 3D) cells and luciferase activities were measured. As shown in Figure 3C and 3D, WT1 repressed the activity of the proximal −403 bp E-cadherin promoter by 5-fold in PC3 cells and 2-fold in DU145 cells. These results confirmed that WT1 regulated the activity of the E-cadherin proximal promoter at the transcriptional level, but did not locate the specific WT1 binding sites involved.

**Figure 4:**
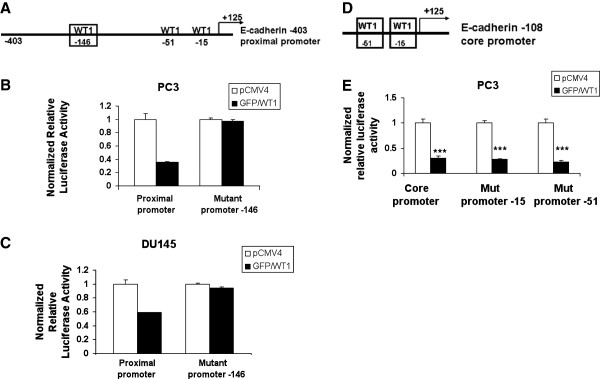
**WT1 binding site located at −146 bp is required for E-cadherin promoter repression.** (**A**) Schematic diagram of the E-cadherin proximal promoter showing the −146 bp WT1 binding site (box). (**B**) PC3 and (**C**) DU145 cells were transiently cotransfected with GFP/WT1 and either with wild type or mutant proximal promoter containing a mutated −146 WT1 binding site. (**D**) Schematic diagram of the E-cadherin core promoter showing −15 and −51 bp WT1 binding sites (boxes). (**E**) PC3 cells were transiently cotransfected with GFP/WT1 and either with wild type or mutant core promoters containing a mutated −15 or −51 WT1 binding site. Luciferase activity was measured and normalized as described in Figure 3. Experiments were repeated three times in triplicate. Data are reported relative to luciferase activity of pCMV4**.** Significance was determined by student’s t-test comparing GFP/WT1 transfected cells relative to pCMV4 transfected cells (***p ≤ 0.001) in three independent experiments.

Since three potential WT1 binding sites were identified in the E-cadherin promoter, it was necessary to determine which one mediated the decreased activity of the E-cadherin promoter. To determine whether the WT1 binding site at -146bp was responsible for the E-cadherin promoter repression, site directed mutagenesis was performed on the −403 proximal promoter (Figure 4A), as described in methods, using primers containing the altered sequence (Table 1). Cotransfection assays of the proximal promoter construct with the mutated −146 site, showed that mutation of the WT1 binding site at-146 bp, eliminated the GFP/WT1 mediated repression of the E-cadherin promoter activity in PC3 cells (Figure 4B). These results were confirmed in another PCa cell line, DU145, (Figure 4C) and showed that the −146 bp WT1 binding site was required for the repression of the proximal E-cadherin promoter by WT1. To determine whether the additional WT1 binding sites located within the G-rich core promoter might also contribute to WT1 mediated repression of the E-cadherin promoter, site-directed mutagenesis was performed as described above. Primers containing the altered sequences for either the −15 or −51 bp WT1 binding sites (Figure 4D) were designed (Table 1). The mutant core promoter constructs were transiently cotransfected along with GFP/WT1 expression construct and luciferase activity was measured. Despite mutation at either the −15 bp or the -51bp WT1 binding sites in the −108 E-cadherin core promoter construct, co-transfection with GFP/WT1 construct, fully repressed the E-cadherin promoter activity (Figure 4E). These results showed that neither of these two WT1 binding sites in the core promoter were required for repression of the E-cadherin promoter by WT1. Taken together, these results suggest that the WT1 binding sites in the core promoter do not contribute to the repression of E-cadherin promoter activity by WT1, rather, the −146 bp site in the proximal promoter mediates E-cadherin expression in PCa cells.

**Table 1:** Primer sets used in site-directed mutagenesis of the E-cadherin promoter

**Site mutated**	**Sequence**
-15 bp ^(a)^	F 5^′^ –CTGGCTGCAGCCACGCA**TTT**CCTCTCAGTGGCGTC-3′
	R 5^′^ – GACGCCACTGAGAGG**AAA**TGCGTGGCTGCAGCCAG-3′
-51bp ^(a)^	F 5^′^- CAATCAGCGGTACGG**TTT**GCGGTGCTCCGGGGC-3′
	R 5^′^- GCCCCGGAGCACCGC**AAA**CCGTACCGCTGATTG-3′
-146bp ^(b)^	F 5^′^- CGTCTATGCGAGGCCG**TTTGTT**CGGGCCGTCAGCTCCG-3′
	R 5^′^- CGGAGCTGACGGCCCG**AACAAA**CGGCCTCGCATAGACG-3′

^(a)^ Primers used to mutate the −15 and −51 bp WT1 binding sites on the E-cadherin −108 core promoter.

^(b)^ Primers used to mutate the −146 bp WT1 binding site on the E-cadherin −403 proximal promoter.

### WT1 alters migration of PCa cells

**Figure 5:**
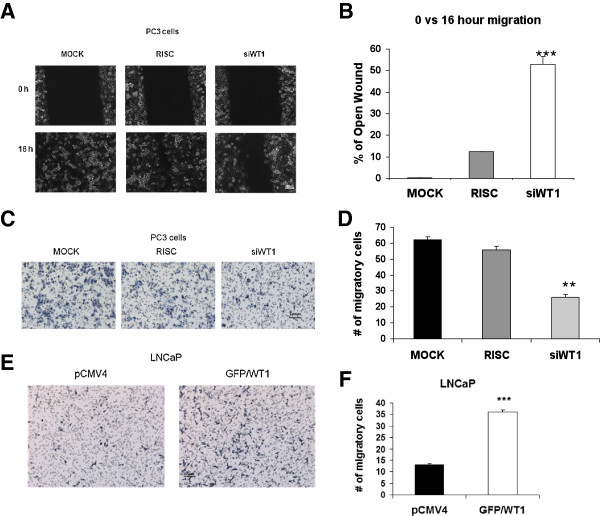
**WT1 affects PCa cell migration.** (**A**) A wound healing assay was performed after transfection with siWT1 RNA or RISC oligonucleotides or MOCK in PC3 cells. Migration potential was measured as described in methods. Monolayers were examined with an Olympus 1 × 70 microscope at 100X magnification and photographed at 0 and 16 hours after wounding. Representative images are shown and the bar represents 100 μM. (**B**) Six images per treatment were measured and analyzed by TScratch software (39) comparing open area at 16 hours to that at 0 hours. Values are presented as percent of open area of monolayers remaining at 16 hours compared to 0 hours and significance was determined by student t-test (**p ≤ 0.01,***p ≤ 0.001) in two independent experiments. (**C**) PC3 cells were transfected as described in (**A**) and placed in the upper chamber and allowed to migrate for 6 hours as described in methods. Representative images of hematoxylin stained membranes containing migratory cells are shown at 100x magnification. (**D**) Migratory cells were counted in six images per treatment and data are presented as average number of migratory cells per chamber. (**E**) LNCaP cells were transfected with pCMV4 or GFP/WT1 expression construct as described in Figure 1. Transfected cells were placed in the upper chamber and allowed to migrate for 48 hours as described in methods. (**F**) Data are presented as per panel D and significance was determined by student t-test (**p ≤ 0.01,***p ≤ 0.001) comparing siWT1 RNA transfected relative to RISC transfected PC3 cells (**D**) and GFP/WT1 transfected relative to pCMV4 transfected LNCaP cells (**F**) in two independent experiments.

Having identified the WT1 site that mediated the repression of E-cadherin, a cell adhesion molecule involved in migration, it was necessary to test the effect of WT1 on cell migration. Silencing of WT1 expression has been shown to inhibit migration of human umbilical vascular endothelial (HUVECs) cells [35]. Since knock-down of WT1 increased E-cadherin mRNA levels in PCa cells (Figure 1E and 1F), and E-cadherin is lost during EMT when cells increase their migratory potential, we tested the affect of WT1 on cell migration. A wound-healing assay was carried out in PC3 cells transfected with either RISC or siWT1 RNA oligonucleotides or MOCK transfected. The confluent monolayer was scratched 72 hours after transfection and cells were allowed to migrate for 16 hours. Six pictures were taken at 0 and 16 hours after transfection (Figure 5A) and images analyzed by T-Scratch software [39] to determine the percentage of wound remaining open at 16 hours compared to 0 hour. The results were reproduced twice and showed that siWT1 RNA oligonucleotides decreased the migration of PC3 cells 4.4-fold compared to RISC control transfection (Figure 5B). Representative samples of RISC control or siWT1 RNA transfected cells were analyzed by qRT-PCR and E-cadherin mRNA levels were increased by 1.9-fold when WT1 was knocked down by 83% (data not shown). Elevated E-cadherin expression in siWT1 RNA transfected cells was consistent with the reduced migration observed.

To validate the wound healing assay results, PC3 cells were transfected with siWT1 RNA or RISC oligonucleotides or MOCK transfected, and after 72 hours, cells were harvested for a transwell migration assay. Cells were allowed to migrate for 6 hours, then six images of stained inserts were taken (Figure 5C) and numbers of cells that had migrated through the insert pores were counted. Results showed that silencing WT1 decreased the average number of migrating PC3 cells by 50%, compared with RISC transfected, and by 60% compared with MOCK (Figure 5D). Representative samples were analyzed by qRT-PCR and E-cadherin mRNA levels were increased by two-fold when WT1 was knocked down by 88% (data not shown). Since siWT1 RNA decreased the migration of PC3 cells, we asked whether the converse, overexpression of WT1, would increase migration of LNCaP cells. LNCaP cells were transfected with pCMV4 or GFP/WT1 expression vectors and after 48 hours, cells were harvested for transwell migration assay as described. Cells were allowed to migrate for 48 hours before inserts were removed, processed and six photographs taken (Figure 5E) to calculate averages for pCMV4 transfected or GFP/WT1 transfected LNCaP cells (Figure 5F). The results showed that overexpression of WT1 increased migration of LNCaP by 3-fold compared with pCMV4 transfected LNCaP cells. These results suggested that WT1 expression was required for the high migratory potential of PC3 cells and loss of WT1 dampened cell migration. Conversely, increased expression of WT1 was sufficient to enhance the low migratory potential of LNCaP cells. Overall these findings link WT1’s role in repression of E-cadherin expression to suppression of cell-cell adhesion and increased migratory potential. Evidence that WT1 mediated repression by binding the E-cadherin promoter *in vivo* and transcriptionally regulated the proximal promoter *in vitro* supports the importance of WT1 in PCa cell migration. Thus, overexpression of WT1 both suppresses expression of the adhesion molecule, E-cadherin, and enhances cell migration.

## Discussion

In the present study we have provided evidence in support of WT1 transcriptionally repressing E-cadherin in PCa cells. First, we observed an inverse relationship of WT1 and E-cadherin mRNA levels in PC3 and LNCaP cells. Secondly, overexpression of WT1 decreased E-cadherin mRNA levels; and suppression of WT1 expression increased E-cadherin mRNA levels. The titration of WT1 levels by transfection altered both E-cadherin expression and the ability of these cells to migrate. Overexpression of WT1 -KTS isoform, and not other WT1 isoforms, has also been proven to increase migration and invasion in human ovarian cancer cells [36]. Finally, the mechanism whereby WT1 was able to repress E-cadherin promoter activity in PCa cell lines was examined. We identified two new potentialWT1 binding sites in the E-cadherin promoter and found that WT1 bound to the E-cadherin promoter in chromatin of both PC3 and LNCaP cells *in vivo*. Specifically, we examined a 220 bp region predicted to have three WT1 binding sites. Although the WT1 binding sites were too closely spaced to resolve single site binding, using site-directed mutagenesis we demonstrated the requirement of the newly identified −146 bp WT1 binding site for the repression of the proximal E-cadherin promoter. Overall, our study shows that WT1 is sufficient to regulate E-cadherin mRNA levels, and our knock-down results demonstrate the necessity of WT1 for regulated E-cadherin expression in PCa cells. Importantly, the biological effect of WT1 mediated changes in E-cadherin levels in these prostate cancer cells lines was altered migration, a key step in metastasis.

One of the hallmarks of cancer is the ability to invade and metastasize [37], traits that require the EMT process and E-cadherin is one of the most important epithelial markers lost in EMT. In the present study, we have shown higher E-cadherin mRNA levels in LNCaP cells compared to PC3 cells, and this is similar to reported levels of E-cadherin protein expression in the same cell lines [31]. In contrast to E-cadherin levels, our results showed that WT1 mRNA levels are higher in PC3 compared to LNCaP cells. Thus, the inverse relationship between WT1 and E-cadherin in LNCaP and PC3 cells lines is consistent with the differences in the aggressive phenotype between these two cell lines [38]. Our evidence that WT1 transcriptionally repressed E-cadherin in PCa cells, led us to examine the functional role of WT1 in migration of PCa cells. Our findings that overexpression of WT1 in LNCaP cells increased their migration potential, is in agreement with those reported in an ovarian cancer cell line [36] where the constitutive expression of the WT1 A isoform promoted cell migration and invasion. However, that study was focused on the effect of WT1 on the cell cytoskeleton, not on cell adhesion molecules such as E-cadherin. Migration is dependent upon disruption of CAMs including integrins, selectins, members of the immunoglobulin superfamily and cadherins. Through their interactions with catenins, cadherins form a junctional complex with cytoskeletal structures such as F-actin. Thus, WT1 may affect migration by regulating components of the junctional complex.

Not only did we find that WT1 over-expression was sufficient to increase migration of LNCaP cells, but inhibition of WT1 expression significantly reduced motility of highly migratory PC3 cells. These results showing that suppression of WT1 by siWT1 RNA oligonucleotide transfection reduced migration of PC3 cells in transwell migration assays, were confirmed by a wound-healing assay using the same siWT1 RNA. This is in agreement with another study showing that silencing of WT1 [35] in human umbilical vascular endothelial cells (HUVECs) inhibited cell migration. However, in HUVECs it appeared that WT1 regulated the ETS-1 (E-twenty six) gene, a transcription factor involved in angiogenesis and invasion. Thus our results in PCa cells suggested a different mechanism whereby WT1 enhanced migration directly through its effect on E-cadherin transcription. This is the first report to show that WT1 alters migration of PCa cells while regulating one of the most important molecules involved in the EMT process, E-cadherin. Taken together, overexpression and silencing of WT1 significantly affected PCa cell migration, supporting our hypothesis that WT1 could behave as an oncogene and promote the process of PCa metastasis.

The mechanism whereby WT1 enhances migration is most likely mediated through transcriptional repression of E-cadherin. WT1 mediated regulation of E-cadherin had been previously described [20,21]. In the study done in murine fibroblasts, WT1 mediated upregulation of E-cadherin in the murine and human E-cadherin promoter [20]. In contrast, our results showed WT1 mediated downregulation of E-cadherin human promoter. This discrepancy could be explained by cell specificity since their study was done in normal murine fibroblast cells, while we used human epithelial cancer cells, which differ in their expression of cadherins. In agreement with our findings, Martinez-Estrada *et al*. [21]has shown downregulation of E-cadherin mRNA and promoter activity in murine cardiac epithelial cells overexpressing WT1. However, they also showed that WT1 mediated indirect downregulation of E-cadherin through the repressor Snail, which we did not find in PCa cells, possibly reflecting differences between normal murine cardiac epithelial cells vs human PCa epithelial cells. Moreover, repression of E-cadherin in cardiac epithelial cells was mediated via the core promoter (at -51bp WT1 binding site). In contrast, our results showed that E-cadherin regulation was mediated through the proximal promoter containing the newly identified WT1 binding site located at −146 bp upstream from the transcription start site. We have shown that WT1 repressed activity of the E-cadherin proximal promoter in both PC3 and DU145 cells, and mutation of the −146 bp WT1 binding site prevented this repression, while mutation of sites in the core promoter had no effect. Taken together, these results suggest that the WT1 binding sites in the core promoter do not contribute to the repression of E-cadherin promoter activity by WT1, rather, the −146 bp site in the proximal promoter is essential for WT1 mediated E-cadherin repression in PCa cells. This is the first report of the newly identified functional WT1 binding site responsible for E-cadherin transcriptional repression and the first demonstrating the effect of WT1 on migration of PCa cells. Although WT1 repressed expression of E-cadherin, a gene that must be suppressed prior to cancer cell migration and is lost during EMT, it is not clear to what extent WT1 contributes to EMT or metastasis of prostate cancer cells. Certainly elevated WT1 expression in prostate cancer compared to normal prostate and BPH is consistent with its role as a regulator of key steps in EMT and migration. Other reports demonstrating a role for WT1 in EMT during epicardial development suggest that its reactivation in prostate cancer epithelial cells may also be associated with EMT in cancer. Thus, WT1 appears to be linked to EMT, migration and possibly metastasis of prostate cancer cells. Since metastasis is a very complicated process that is still poorly understood, further studies are needed to determine how WT1 is involved in EMT and metastatic processes of PCa cells.

## Conclusions

We have shown that WT1 bound the E-cadherin promoter *in vivo*; WT1 overexpression decreased, and WT1 silencing increased, E-cadherin mRNA levels. Moreover WT1 decreased E-cadherin promoter activity and the −146 bp binding site was required for this decrease. Importantly, increased WT1 expression enhanced migration of LNCaP cells and silencing WT1 decreased migration of PC3 cells. Thus, we conclude that WT1 both regulates E-cadherin levels and contributes to the migratory potential of prostate cancer cells. These data are consistent with an oncogenic function for WT1, enhancing migration and metastasis of prostate cancer cells.

## Methods

### Cell lines and reagents

The LNCaP, PC3, and DU145 PCa cells and RWPE-1 non-neoplastic cells were obtained from the American Type Culture Collection (ATCC, Manassas,VA). LNCaP cells were grown in RPMI-1640 media supplemented with 10% Fetal Calf Serum (FCS) and antibiotics, while PC3 and DU145 cells were grown in DME-F12 media with the same supplement. RWPE-1 cells were grown in K-SFM supplemented with 0.05 mg/ml bovine pituitary extract and 5 ng/ml EGF. All cells were maintained in 5% CO_2_ at 37°C.

### Transfection

For overexpression of WT1, LNCaP and PC3 cells were grown in 35mm tissue culture dishes until 80% confluent. Cells were transfected either with empty expression vector as a control, pCMV4, (Promega, Madison, WI) or the cytomegalovirus (CMV) promoter-driven pGFP/Wt1(A) expression construct encoding the murine Wt1 protein (lacking both KTS insertion and exon 5) fused at the amino terminus to the Green Fluorescent Protein [34]. Cells were transfected with lipofectamine 2000 (Invitrogen, Carlsbad, CA) in a 1:3 DNA/lipid ratio as per manufacturer’s recommendations. The cells were incubated for 5 hours in antibiotic-free media, then the transfection medium was replaced with complete medium and after 48 hours cells were harvested for RNA isolation or migration assays. Murine Wt1 primers were used to confirm overexpression of the murine GFP-Wt1 expression construct (Table 2).

**Table 2:** TaqMan and Sybergreen qRT-PCR primer sets used for expression analyses

**Functional Class**	**Gene**	**Sequence**
Housekeeping gene	18S normalizer ^(a)^	Hs9999990_s1
	GAPDH ^(b)^	F 5^′^CCATCACCATCTTCCAGGAG 3^′^
		R 5^′^ GGATGATGTTCTGGAGAGCG
Transcription Factor	Wt1(mouse) ^(b)^	F 5^′^ TGGTCTGAGCGAGAAAACCT 3^′^
		R 5^′^TCTTCCGAGGCATTCAGGAT 3^′^
	WT1(human) ^(b)^	F 5^′^GAGAGCCAGCCCGCTATTC 3^′^
		R 5^′^ CATGGGATCCTCATGCTTG 3^′^
	Snail(human) ^(b)^	F 5^′^ ACCCCACATCCTTCTCACTG 3^′^
		5^′^ TACAAAAACCCACGCAGACA 3^′^
Adhesion molecule	E-cadherin ^(a)^	Hs00170423_m1

^(a)^ TaqMan probes used to amplify E-cadherin gene.

^(b)^ Sybergreen primers used to amplify murine Wt1 and human WT1 or Snail genes.

For WT1 silencing, LNCaP and PC3 cells were grown in 35mm tissue culture dishes until 50–60% confluent. Cells were transfected with DharmaFECT#2 (Thermo Scientific, Lafayette, CO) and either 50nM RISC (control, non-targeting siRNA oligonucleotides with impaired ability for RISC interaction) or siWT1 RNA specific oligonucleotides designed to target WT1 (Dharmacon) or MOCK (without oligonucleotides or transfection reagent). Initially pools of si RNA oligonucleotides were tested until si WT1 RNA # 7 and #8 were proven efficacious. Primarily, results for silencing by si WT1 RNA oligonucleotide # 7 are shown in this study, but where stated, results were confirmed by si WT1 RNA # 8. The transfections were performed in antibiotic free media as described above, but cells were harvested after 72 hours of treatment for RNA isolation, migration or wound healing assays. Human WT1 primers were used to confirm knock-down of endogenous human WT1 expression by si WT1 RNA (Table 2).

### RNA isolation and quantitative real time PCR

RNA was isolated from confluent cells using the GenElute Mammalian Total RNA Miniprep Kit following the manufacture’s recommendations (Sigma-Aldrich, St Louis, MO). RNA concentrations were measured in a NanoDrop ND-1000 Spectrophotometer (Nanodrop Technologies, Inc, Wilmington, DE) and 1μg RNA was reverse transcribed using High Capacity cDNA Reverse Transcription Kit (Applied Biosystems ABI, Foster City, CA).

Quantitative Real Time PCR (qRT-PCR) was performed in triplicate using TaqMan Universal Master Mix (ABI, Foster City, CA) and E-cadherin and 18S normalizer TaqMan human probes (Table 2). Ten nanograms of cDNA samples were amplified using an ABI 7000 thermocycler. Amplification conditions were 95°C for 10 minutes, and 40 cycles of 95°C for 15 seconds and 60°C for 1 minute. After normalizing E-cadherin to 18S gene expression, the comparative Ct method was used to analyze gene expression differences between cells transfected with pCMV4 or GFP/Wt1. Student t-test was used to analyze the significance.

qRT-PCR was also performed using Brilliant II Fast Syber Green Master Mix (Agilent Technologies, La Jolla, CA) and GAPDH (normalizer), Wt1, WT1 or Snail primers (Table 2). Each sample was assayed in triplicate as described above, but using Stratagene 3000MxPro thermocycler (Agilent Technologies, La Jolla, CA). Amplification conditions for WT1 were 95°C for 2 minutes and 40 cycles of 95°C for 5 seconds and 60°C for 20 seconds. Data were analyzed as described above, but normalizing to GAPDH and comparing cells transfected with RISC to those transfected with siWT1 RNA oligonucleotides. Student t-test was used to analyze the significance.

### Chromatin immunoprecipitation (ChIP)

LNCaP and PC3 cells were grown until ~80% confluent and then transfected with a GFP/Wt1 expression construct as described above. After 48 hours, cells were harvested for chromatin analysis as recommended by manufacturer, (Millipore EZ-Magna ChIP, Temecula, CA) and described previously [33]. After cross-linking with a 1% formaldehyde solution (pH 4), cells were then washed with cold PBS containing protease inhibitors cocktail (Millipore, Temecula, CA). Chromatin was sheared by sonication at medium power using six cycles of 10 seconds of sonication followed by 30 seconds on ice using a Microson Ultrasonic Cell Disruptor (Misonix Incorporated, Farmingdale, NY). Sheared chromatin was analyzed by gel electrophoresis and chromatin of 200–1000 bp was tested. Sheared chromatin was suspended in protein G magnetic beads with either 5 μg of anti-SP1 or 1 μg of anti-Pol II antibodies (as positive controls) or 1 μg of mouse IgG fraction (negative control) (Millipore, Temecula, CA) or a mixture of anti-WT1 antibodies containing both C19 and N18 antibodies (Santa Cruz Technologies, Santa Cruz, CA) and rocked overnight at 4°C. The magnetic beads-Ab-protein-chromatin complexes were washed and incubated with proteinase K as per manufacturer’s recommendation (Millipore, Temecula, CA). Eluted chromatin was purified using DNA purification columns and chromatin was amplified using specific E-cadherin primers (−180 Forward 5^′^-AACTCCAGGCTAGAGGGTCA-3^′^; +31 Reverse 5^′^-TCACAGGTGCTTTGCAGTTC-3^′^) that flanked three potential WT1 binding sites in the promoter region of E-cadherin. Endpoint PCR was performed using the following PCR conditions: 94°C for 2 minutes, 34 cycles of 94°C for 20 seconds, 59°C for 30 seconds, 72°C for 30 seconds and final extension at 72°C for 2 minutes. The 210 bp PCR products were separated by electrophoresis and visualized by ethidium bromide in a 1% agarose gel. Validation of the endpoint PCR was done by Sybergreen qRT-PCR as described above, using same primers as for endpoint PCR. All ChIP experiments were done at least twice with different chromatin preparations and tested in two different cell lines. To confirm specificity of WT1 binding, amplification of immunoprecipitated chromatin was tested by negative control primers located approximately 1 kb from the transcription start site (−1015 Forward 5^′^-ACGCCTGTAATCCAACACTTCAGG-3^′^ and −714 Reverse 5-AAATTAGGCTGCTAGCTCAGTGGC-3^′^) and flanking a region devoid of potential WT1 binding sites.

### Luciferase assays

Cells were transfected in a 1:3 DNA/lipid ratio as described above. For empty vector control wells, 750 ng of pCMV4 (Promega, Madison WI) was used along with 250 ng of E-cadherin luciferase construct. For experimental wells, 500 ng of GFP/Wt1 expression construct along with 250 ng of E-cadherin luciferase construct and 250 ng of empty vector expression construct pCMV4 was used (to bring total DNA to 1μg per well). After 48 hours cells were harvested for measurement of luciferase activity using passive lysis buffer (Promega, Madison WI). Lysates were centrifuged and the supernatant was analyzed using luciferase assay system (Promega, Madison WI) and activity was measured in a Turner luminometer 20/20 (Turner Biosystems, Sunnyvale, CA). Protein concentrations were calculated using bicinchoninic acid (BCA) method as described (Thermo Scientific Pierce, Rockford, IL). Promoter activity was normalized to protein concentration for each well and represented by relative light units (RLU) of luciferase activity. Experiments were performed in triplicate and reproduced at least 3 times.

### Cloning of the E-cadherin proximal promoter construct

The core (−108 + 125) E-cadherin promoter reporter construct was purchased from Addgene (Cambridge, MA), however, the larger proximal (−403 + 125) E-cadherin promoter was cloned in the PGL3 basic luciferase vector (Promega, Madison WI) using PCR amplified DNA. The primer sequences used were: Forward 5^′^ - GAGGTACCAGTGAGCTGTGATCGCAC-3^′^ and Reverse 5^′^ -GGAGCTCGAACTGACTTCCGCAAGCTC-3′. The forward primer contained the *KpnI* site and the reverse primer contained the *SacI* site. Endpoint PCR was performed using Applied Biosystems reagents, as described for ChIP analysis, except that 5% DMSO was added to reduce secondary structures in GC-rich DNA. The following PCR conditions were used: 95°C for 5 minutes, 35 cycles of 95°C for 1 minute, 62°C for 1 minute, 72°C for 1 minute and final extension at 72°C for 5 minutes. Both the PGL3 basic luciferase vector and purified PCR products were digested with *KpnI* and *SacI* restriction enzymes, ligated, then after transformation, the identity of the purified plasmid was confirmed by sequencing. Activity of E-cadherin promoter constructs was tested by transfection and luciferase assays as described above.

### Site directed mutagenesis

Several WT1 binding sites in the E-cadherin promoter were mutated using the QuickChange Site-Directed Mutagenesis Kit (Stratagene, Agilent Technologies, Santa Clara, CA) and primers containing the desired mutations (shown in Table 1). PCR was performed using either the E-cadherin core (−108) or proximal (−403) reporter constructs as templates along with the appropriate mutant primers and 5% DMSO (to reduce secondary structures). The following PCR conditions were used: 95°C for 30 seconds, 18 cycles of 95°C for 30 seconds, 68°C for 1 minute, 68°C for 6 minutes. After amplification, parental strands were digested by *DpnI,* which degrades parental methylated DNA, and XL1-Blue supercompetent cells were transformed with newly synthesized mutant DNA. Plasmid DNA was isolated, purified (Qiagen, Valencia, CA) and sequenced to verify that the correct base pairs were changed. The effect of each mutation was tested by luciferase assays as described earlier.

### Migration assay

PC3 cells were transfected with either RISC or siWT1 RNA oligonucleotides or MOCK as described above. LNCaP cells were transfected with either pCMV4 empty vector or GFP/Wt1 expression construct as described above. After 72 hours (PC3 cells) or 48 hours (LNCaP cells) of incubation, migration assays were performed using ThinCerts migration inserts with 8μm pore size (Bioexpress, Kaysville, UT). Briefly, 2 × 10^5^ transfected cells in serum free media were added to the top chamber of the insert, while the bottom chamber contained media with 10% FBS providing the chemoattractant signal. The cells were allowed to migrate for 6 hours (PC3 cells) or 48 hours (LNCaP cells), then inserts were removed and the remaining non- migrating cells on the upper surface of the membrane were removed with a cotton swab. The cells that migrated to the lower surface of the membrane were fixed with 4% formaldehyde and stained with Harris Hematoxylin Solution (Sigma Aldrich, St. Louis, MO). After washing, the membrane was peeled off the plastic inserts, placed on glass microscope slides and mounted using HistoChoice mountaing media (Amresco, Solon, OH). Migrating cells were examined by microscopy at 200X magnification with Olympus 1 × 70 microscope (Center Valley, PA), then pictures were taken of six different randomly selected fields and migrating cells were counted manually. The average number of migratory cells for each transfection condition was calculated. T-test was used to determine significance. Migration assays in both cell lines were reproduced twice. Additionally, to verify efficacy of siWT1 RNA oligonucleotides, RNA was also collected from representative samples of the above transfected PC3 cells and qRT-PCR analysis of both WT1 and E-cadherin expression was done as described above.

### Wound healing assay

PC3 cells were transfected with either RISC or siWT1 RNA oligonucleotides or MOCK as described above. After 72 hours of treatment, transfected cells formed a confluent monolayer and a 200μl pipette tip was used to scratch a “wound” into the confluent monolayer. Pictures were taken as described above, but at 100X magnification, at both 0 and 16 hours after the “wounding” to provide a comparison of migration over time. TScratch software [39] was used to analyze six images, measuring the differences in migration between MOCK, RISC and siWT1 RNA oligonucleotides transfected cells. Values were calculated as percentage (%) of open area (“wound”) remaining at 16 hours compared to 0 hour. T-test was performed to determine significance. Wound healing assays in PC3 cells were reproduced twice with similar results. As described above, to verify efficacy of siWT1 RNA oligonucleotides, RNA was also collected from representative samples of the above transfected PC3 cells and qRT-PCR analysis of both WT1 and E-cadherin expression was done as described above. Due to irregular growth of LNCaP monolayers, analyses by TScratch software was not attempted for GFP/Wt1 transfected LNCaP cells, thus migration data in LNCaP cells was not confirmed by wound healing assays.

## Abbreviations

WT1: Wilms’ tumor gene; PCa: Prostate cancer; EMT: Epithelial to mesenchymal transition; GFP: Green fluorescent protein; ChIP: Chromatin Immunoprecipitation; FCS: Fetal calf serum; siRNA: Short-interfering RNA; qRT-PCR: Quantitative real time PCR.

## Competing interests

The authors declare no potential conflicts of interest with respect to the research, authorship and/or publication of this article.

## Authors’ contributions

AB acquired data and performed the statistical analysis. SP assisted with qRT-PCR assays, revised and approved manuscript. AB and GF conceived and designed study; analyzed and interpreted data; drafted, revised and approved manuscript. All authors read and approved the final manuscript.

## Supplementary Material

Additional file 1 **Figure S1.** Top Panel: Positions of potential WT1 binding sties are listed and arrows indicate location of PCR primers for amplification of chromatin. Bottom two panels: ChIP assays were performed with chromatin from PC3 (left) and LNCaP (right) cells. Cells were transfected with GFP/WT1 construct and harvested after 48 hours. Chromatin was crosslinked and then immunoprecipitated with either IgG (negative control), WT1 or SP1 antibodies. Input or immunoprecipitated DNA was amplified by endpoint PCR using primers, shown as arrows in top panel, that amplify a 300 bp region devoid of potential WT1 binding sites and are located ~ 1Kb upstream of the transcriptional start site. Amplified products were analyzed by gel electrophoresis and representative images are shown. Click here for file
