# Supplementary figures and images for: The Wilms’ tumor gene (WT1) regulates E-cadherin expression and migration of prostate cancer cells
Source: Mol Cancer. 2013 Jan 8;12:3. doi: 10.1186/1476-4598-12-3 (PMC3568020; doi:10.1186/1476-4598-12-3)

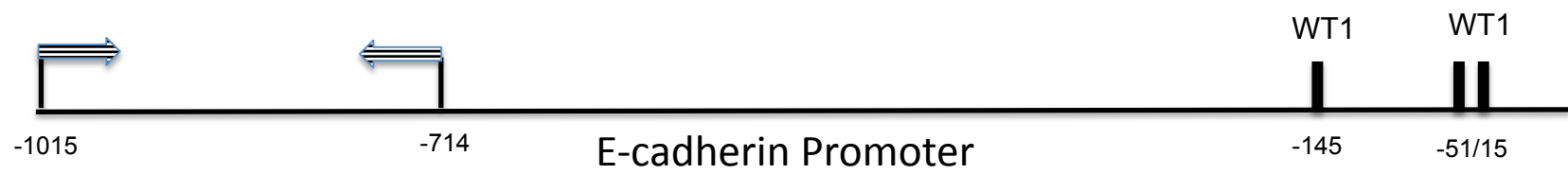

**PC3**

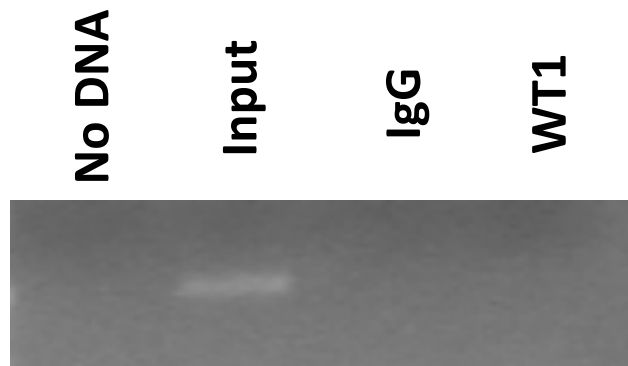

**LNCaP**

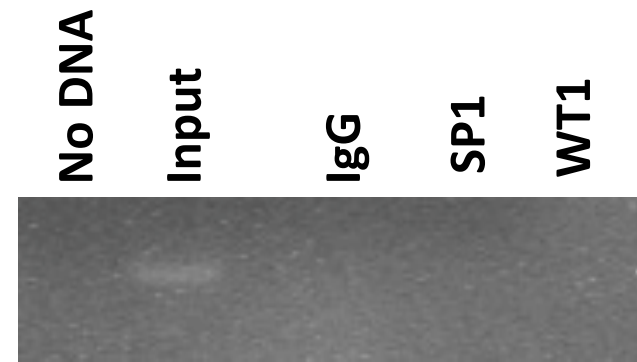

Supplemental Figure 1

Supplement: Additional file 1 — Figure S1. Top Panel: Positions of potential WT1 binding sties are listed and arrows indicate location of PCR primers for amplification of chromatin. Bottom two panels: ChIP assays were performed with chromatin from PC3 (left) and LNCaP (right) cells. Cells were transfected with GFP/WT1 construct and harvested after 48 hours. Chromatin was crosslinked and then immunoprecipitated with either IgG (negative control), WT1 or SP1 antibodies. Input or immunoprecipitated DNA was amplified by endpoint PCR using primers, shown as arrows in top panel, that amplify a 300 bp region devoid of potential WT1 binding sites and are located ~ 1Kb upstream of the transcriptional start site. Amplified products were analyzed by gel electrophoresis and representative images are shown. [file 1476-4598-12-3-S1.pdf]
